# Specific Microbial Communities Associate with the Rhizosphere of *Welwitschia mirabilis*, a Living Fossil

**DOI:** 10.1371/journal.pone.0153353

**Published:** 2016-04-11

**Authors:** Angel Valverde, Pieter De Maayer, Tanzelle Oberholster, Joh Henschel, Michele K. Louw, Don Cowan

**Affiliations:** 1 Department of Genetics, Centre for Microbial Ecology and Genomics, University of Pretoria, Pretoria, South Africa; 2 Namib Ecological Restoration and Monitoring Unit, Gobabeb Research and Training Centre, Walvis Bay, Namibia; 3 SAEON Arid Lands Node, Hadison Park, Kimberly, South Africa; 4 Swakop Uranium, Swakopmund, Namibia; University of Milan, ITALY

## Abstract

*Welwitschia mirabilis* is an ancient and rare plant distributed along the western coast of Namibia and Angola. Several aspects of *Welwitschia* biology and ecology have been investigated, but very little is known about the microbial communities associated with this plant. This study reports on the bacterial and fungal communities inhabiting the rhizosphere of *W*. *mirabilis* and the surrounding bulk soil. Rhizosphere communities were dominated by sequences of Alphaproteobacteria and Euromycetes, while Actinobacteria, Alphaproteobacteria, and fungi of the class Dothideomycetes jointly dominated bulk soil communities. Although microbial communities within the rhizosphere and soil samples were highly variable, very few “species” (OTUs defined at a 97% identity cut-off) were shared between these two environments. There was a small ‘core’ rhizosphere bacterial community (formed by *Nitratireductor*, *Steroidobacter*, *Pseudonocardia* and three *Phylobacteriaceae*) that together with *Rhizophagus*, an arbuscular mycorrhizal fungus, and other putative plant growth-promoting microbes may interact synergistically to promote *Welwitschia* growth.

## Introduction

Plants live in association with a great number of microorganisms [1], the so-called plant microbiome. The plant microbiome can have profound effects on seed germination, seedling vigour, plant growth and development, nutrition, diseases and productivity [2]. In return, plants secrete a wide range of compounds, including sugars, vitamins, amino acids, purines and nucleosides [3] that support microbial communities and influence their composition and activities [4]. The rhizosphere, the narrow zone of soil that surrounds and is influenced by plant roots, is a microbial diversity hotspot [2, 5]. Recent studies have shown that, in natural ecosystems, plant diversity [6] and the genotypes of individual plants [7] can influence the composition of their root-associated microbes. Yet, more research is still needed to better understand the plant microbiome.

*Welwitschia mirabilis* is an uncommon plant found in the western hyper-arid desert regions of Namibia and southern Angola, where it occurs in isolated populations ranging from 2 to more than 1000 long-living individuals [8]. It is thought that the older specimens may be more than 1500 years old [9]. The plant, which was first discovered in 1859 by the Austrian botanist Friedrich Welwitsch, has always fascinated scientists because of its primitive nature. *W*. *mirabilis* is a monogeneric and monospecific member of the family *Welwitschiaceae* that is grouped with the genus *Ephedra* and *Gnetum* under the plant Division *Gnetophyta* [10], a small group of seed plants that has intermediate characteristics between Angiosperms and Gymnosperms. In addition to its scientific significance, *Welwitschia* is of considerable importance to the local ecosystem because it provides refuge, shade, food, and water to many species of animals that inhabit the Namib [8]. As a result researchers have extensively studied the botany, physiology and ecology of *Welwitschia* plants [11]. However, very little is known about the microbial communities associated with *Welwitschia* (but see [12]).

In this study, we used 454 amplicon pyrosequencing to analyse the bacterial and fungal communities inhabiting the *Welwitschia* rhizosphere and compared them with those from the bulk soil. We asked: Are *Welwitschia* rhizosphere and bulk soil communities phylogenetically distinct, does *W*. *mirabilis* present a core community of rhizosphere microbes?

Given the fact that *Welwitschia* have co-evolved with other organisms for more than 110 million years [13], we would expect *Welwitschia* to have selected for a specific cohort of rhizosphere microbes.

## Materials and Methods

### Sample collection

Sampling of *Welwitschia mirabilis* (S1 Fig), a unique and protected plant (CITES Appendix II), was undertaken in September 2012 at a single location (S22°40’18.84”, E14°51’35.69”) in the Namib Desert, under the auspices of the permission granted to Swakop Uranium to transplant three *Welwitschia* plants, located 5–7 m apart, as part of the construction of a road system to support their mining operation. Because *Welwitschia* roots were found to be embedded in a matrix of calcrete, only 5 rhizosphere soil samples (i.e. soil closely adhering to the root systems, depth 20–30 cm) were obtained from the three plants (approx. 150–300 year old, visual estimation). Five bulk soil samples (i.e. unvegetated soil 10–20 cm distant from the root system, depth 20–30 cm) were also collected. Samples, consisting of *ca*. 20 g of soil, were stored in 50-ml Falcon tubes containing RNALater solution (Sigma-Aldrich, USA) and shipped at room temperature to the Namibian Ministry of Environment and Tourism for delivery to South Africa. All samples were processed within two weeks of collection. Samples were collected under sampling permit number 1653/2011 issued by the Namibian Ministry of Environment and Tourism.

### Soil DNA extraction, fragment amplification and high-throughput sequencing

Total soil DNA was extracted using the MoBio PowerSoil DNA isolation kit (MoBIO, USA) following the manufacturer’s instructions. Partial bacterial 16S rRNA gene amplicons were produced using the primers 27F (5’-AGRGTTTGATCMTGGCTCAG-3’) and 519R (5’- GTNTTACNGCGGCKGCTG-3’), targeting the V1-V3 hypervariable region, as in [14]. Partial fungal ITS amplicons were produced using the primer set ITS1F (5’-CTTGGTCATTTAGAGGAAGTAA-3’) and ITS4 (5’-TCCTCCGCTTATTGATATGC-3’), targeting the hypervariable ITS2 region, as in [15]. PCR was performed using 50 ng soil DNA and the HotStarTaq Plus Master Mix Kit (Qiagen, USA). Amplicon products from different samples were mixed in equal concentrations and purified using Agencourt Ampure beads (Agencourt Bioscience Corporation, USA). Samples were sequenced at the Molecular Research LP next generation sequencing service (http://www.mrdnalab.com) using Roche 454 FLX titanium instruments and reagents and following manufacturer’s guidelines.

### Sequence processing

Quality processing of 16S rRNA gene sequences was performed in Mothur (v.1.35.0) [16] following a previously described pipeline [17]. Briefly, the FASTA quality and flow data were extracted using the *sffinfo* command. Low-quality sequences were removed using *trim*.*flows* and *shhh*.*flows*, which is an implementation of the PyroNoise component of the AmpliconNoise suite of programs [18]. The data set was reduced to only unique sequences using *unique*.*seqs*. An alignment was generated using the *align*.*seqs* command by aligning the data to the SILVA bacterial database. The *screen*.*seqs* command was used to reduce the data to the overlapping region of the sequences. Chimeric sequences were removed through *chimera*.*uchime*. After quality filtering, sequences were used to construct a distance matrix and grouped into OTUs (cut-off level of 97%, species level [19]). The taxonomic affiliations of the OTUs were determined using the naive Bayesian rRNA classifier [20], at an 80% confidence level. Sequences that had the highest similarity to chloroplast sequences were removed prior to further analysis.

Pre-alignment steps for fungal ITS sequences were as described above, but we trimmed reads to a maximum length of 300 bases. Chimeras were eliminated using *chimera*.*uchime*. To cluster unaligned sequences into OTUs, we created a pairwise distance matrix using *pairwise*.*seqs* and then created clusters sharing 97% or greater sequence identity using the *cluster* command. Classification of sequences was performed with *classify*.*otu* using the UNITE ITS reference database (http://unite.ut.ee/repository.php).

The sequence data generated in this study were deposited in the NCBI Sequence Read Archive and are available under the project number SRP061179.

### Statistical analysis

All statistical analyses were conducted in Mothur v.1.35.0 and R v.3.2.0 (R Foundation for Statistical Computing; http://www.R-project.org). Singleton sequences were removed, and each sample was subsampled with the Mothur command *sub*.*sample* to 449 reads for bacterial and 2787 for fungal OTUs, which was the minimum number of sequences remaining in a single sample. Sample rarefaction, as in [14], ensures equal sampling effort across samples. We visualised similarities in community composition using non-metric multidimensional scaling (nMDS) with weighted UniFrac distances. Differences in community structure were assessed by ANOSIM analysis using the *anosim* function in vegan (cran.r-project.org/package = vegan). The number of shared OTUs between communities/samples was visualized using the *venn* function in gplots (cran.r-project.org/package = gplots). The mean fungal and bacterial diversities were compared using paired two-tailed Student’s t-tests. The compositions of major fungal and bacterial genera were compared using UPGMA clustering on Hellinger-transformed Bray-Curtis distances together with a heatmap of abundance data created with *heatmap*.*2* in gplots.

## Results and Discussion

Here we report on the microbial community associated with *Welwitschia mirabilis* roots using metagenomic DNA from rhizosphere (n = 5) and bulk (n = 5) soil. The analysis, after quality filtering and removal of singletons, included a total of 31522 amplicon sequences with an average sequence length of 241 bp for the 16S rRNA gene and 283 bp for the ITS. The number of sequences was lower for the 16S rRNA gene assays (3712 sequences) than for the ITS assays (27810 sequences). 407 bacterial 16S rRNA gene OTUs and 139 ITS OTUs, both at 97% sequence identity, were included in the analysis.

Bacterial richness was significantly greater than fungal richness in both rhizosphere and bulk soil communities (S1 Table), although the number of sequences was 7-fold higher for fungi (S2 Table). A similar result was found in a recent study carried out in the rhizosphere of invasive *Berberis thungerbii* [7]. In general, bulk soil bacterial communities were more diverse than rhizosphere bacterial communities (S1 Table), probably due to the selection process that gradually differentiates the root microbiome from the surrounding soil biome [3]. No differences in diversity were detected between the fungal communities in the two ecosystems (S2 Table), corroborating recent findings suggesting that plant-soil feedbacks do not influence the diversity of soil fungi [21]. These results should be interpreted with caution, as rarefaction curves and Good’s coverage values indicate that we did not sample all members of the bacterial and fungal communities (S2 Table, S2 Fig). However, the goal of this study was not to obtain a full coverage of the diversity in the samples, but rather to use 454-sequencing as a tool to gain taxonomic information and assess beta-diversity patterns.

**Fig 1:**
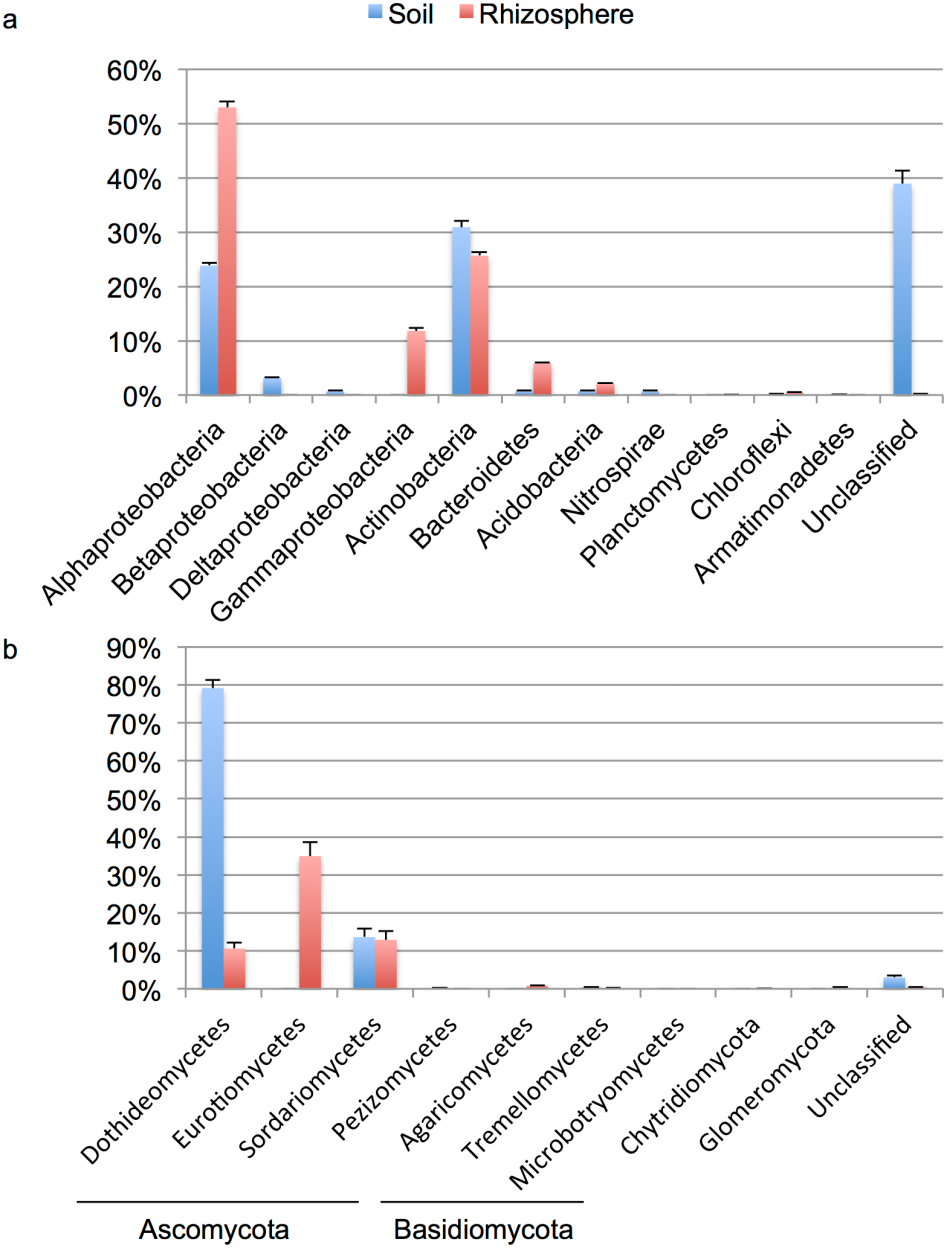
Relative proportions of the (a) bacteria and (b) fungi associated with the *Welwitschia* rhizosphere and bulk soil. Error bars indicate mean ± SE.

**Fig 2:**
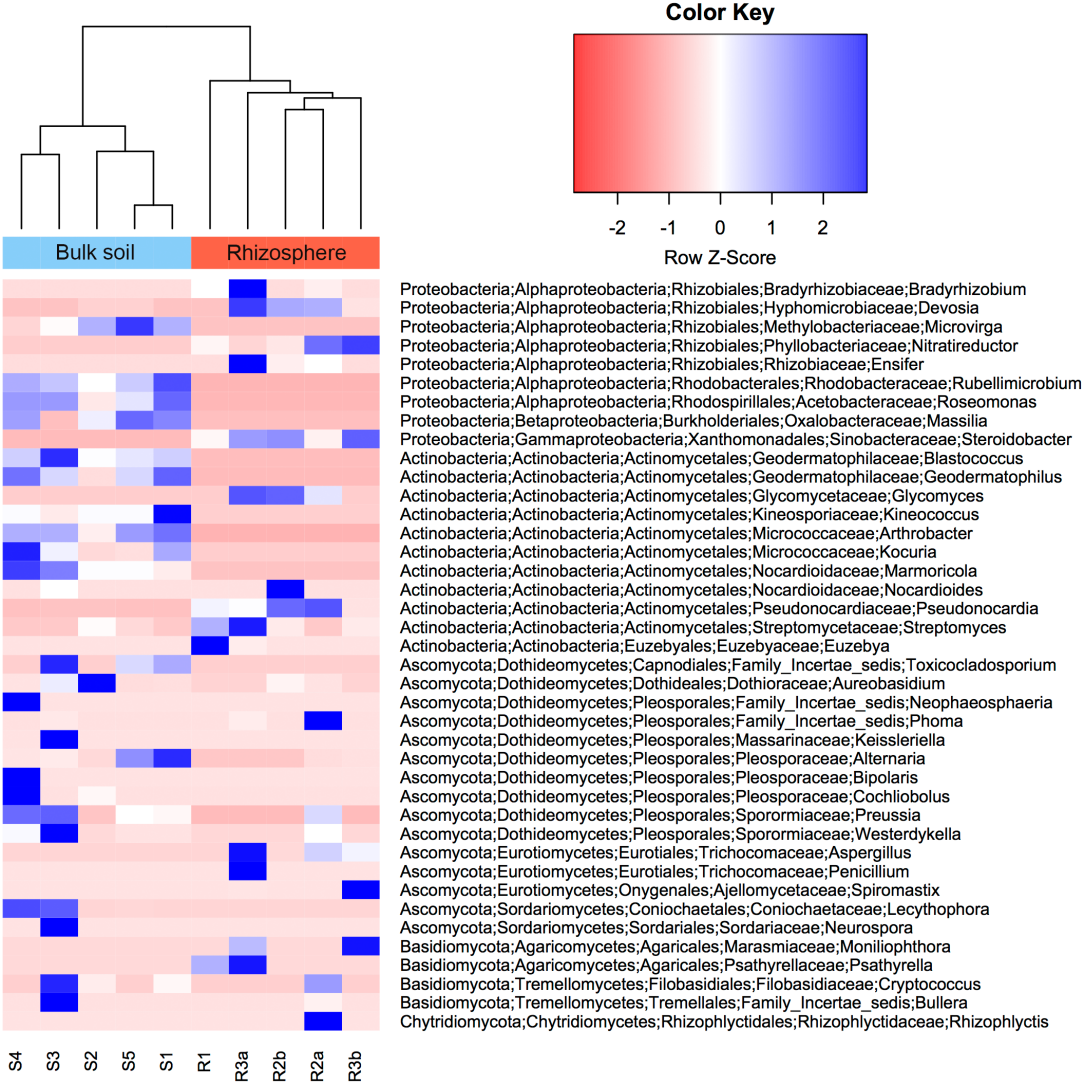
Heatmap displaying the most abundant genera for rhizosphere and bulk soil samples. Samples are clustered based on the percent relative abundance of the forty dominant genera (twenty bacteria and twenty fungal genera) shown as rows in this figure. Taxonomy for each genus is presented in the order: phylum, class, order, family, genus. Sample nomenclature indicates the sample type (S = bulk soil; R = rhizosphere), replicate (S = 1 to 5, R = 1 to 3) and pseudoreplicate (a, b).

The rhizosphere bacterial communities of *W*. *mirabilis* predominantly consisted of sequences from the Proteobacteria (66%, mostly Alphaproteobacteria (53%) and Gammaproteobacteria (12%)), Actinobacteria (26%), Bacteroidetes (6%) and Acidobacteria (2%) (Figs 1a and S3a); these numbers represent the average percentage of sequences across the five samples. Bulk soil bacterial communities were jointly dominated by sequences from the Actinobacteria (31%) and Proteobacteria (28%, Alphaproteobacteria (24%), Betaproteobacteria (3%), Deltaproteobacteria (1%)). Bacteroidetes, Acidobacteria and Nitrospirae each contributed 1%. These phyla have been shown to be widely represented in rhizosphere and soil bacterial communities [7, 22–25], although their relative abundance vary in the different studies. At the genus level, *Nitroreductor* (20%), *Steroidobacter* (12%), *Pseudonocardia* (9%), *Devosia* (4%) and *Glycomyces* (2%) were prevalent in the rhizosphere (Fig 2); whereas *Rubellimicrobium* (9%), *Kocuria* (5%), *Geodermatophilus* (5%) and *Microvirga* (4%) were the genera most frequently found in the bulk soil. Interestingly most genera found in the rhizosphere contain isolates with plant growth-promoting activities (discussed below).

Rhizosphere fungal communities consisted mainly of Ascomycota (98%, Eurotiomycetes (35%), Sordariomycetes (13%), Dothideomycetes (11%)) (Figs 1b and S3b). Basidiomycota, Glomeromycota and Chytridiomycota contributed 1%, 0.3% and 0.1%, respectively. Bulk soil fungi were also dominated by sequences from the Ascomycota (96.6%), but primarily composed by Dothideomycetes (79%) and Sordariomycetes (14%). Basidiomycota represented a discrete 0.4%. This is in contrast to what has been reported in a global study, where Basidiomycota encompassed the largest proportion of sequences [21]. However, to the best of our knowledge, desert soils were not sampled in the later study. At the genus level, *Aspergillus* (19%), *Spiromastix* (16%), *Phoma* (6%) and *Aternaria* (3%) dominated rhizosphere fungal communities (Fig 2); whereas *Alternaria* (28%) and *Lecythophora* (4%) were prevalent in soil fungal communities. *Alternaria* and *Phoma* are plant pathogens, while *Aspergillus*, *Spiromastix* and *Lecythophora* can be classified as saprotrophs (Data file S2 in [21]).

Strikingly, an average of 7% and 39% of the bacterial sequences, for rhizosphere and bulk soil respectively, remained unclassified at the phylum level with the RDP classifier tool (Figs 1a and S3a). For fungi, using the UNITE database, unclassified sequences represented only 0.4% and 3% of the total, for rhizosphere and bulk soil samples respectively (Figs 1b and S3b). The fact that most unclassified sequences were retrieved from bulk soil samples suggests that this ecosystem remains substantially understudied.

**Fig 3:**
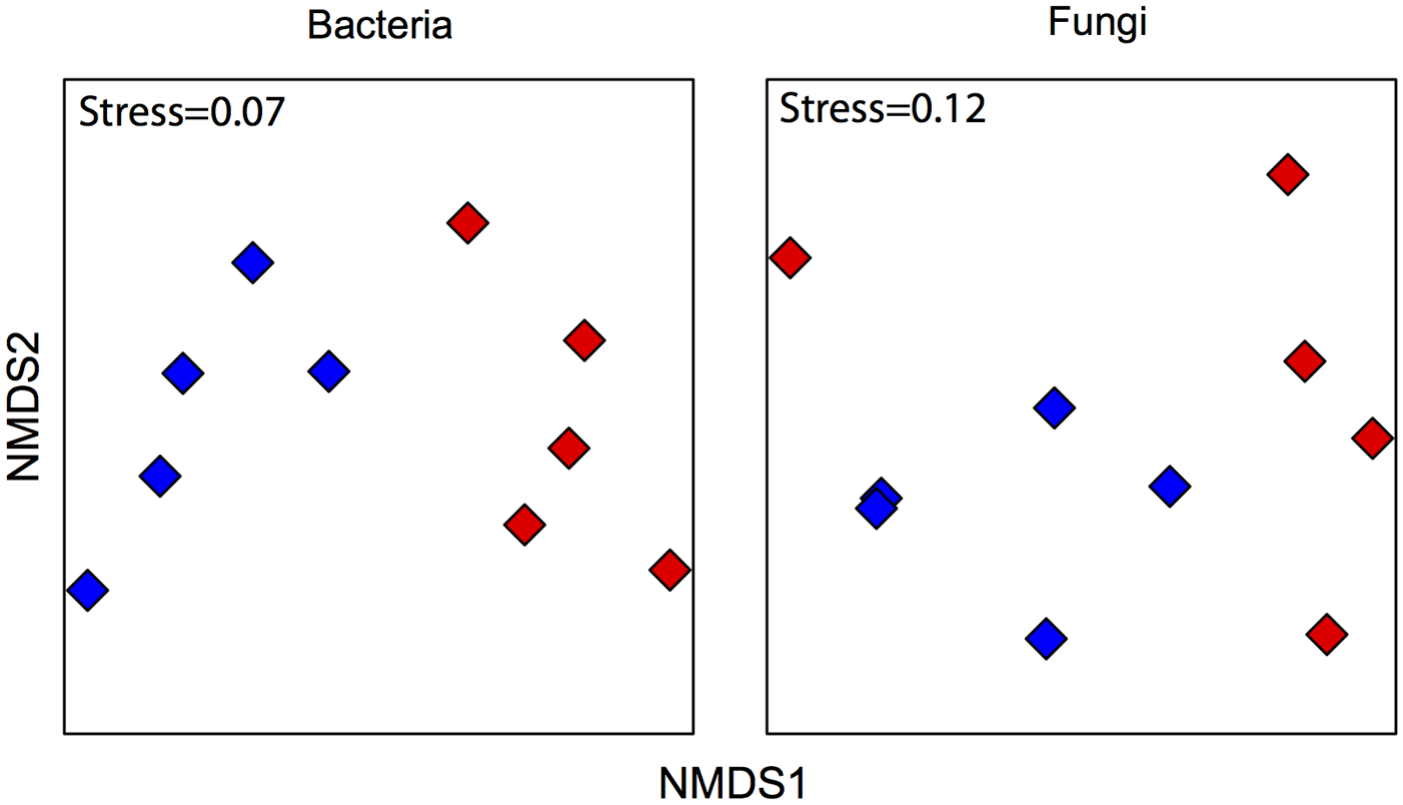
nMDS ordination plot (UniFrac dissimilarity matrix). Each point represents the bacterial or fungal community of an individual sample. Rhizosphere communities are indicated by red diamonds, while bulk soil communities are denoted by blue diamonds.

**Fig 4:**
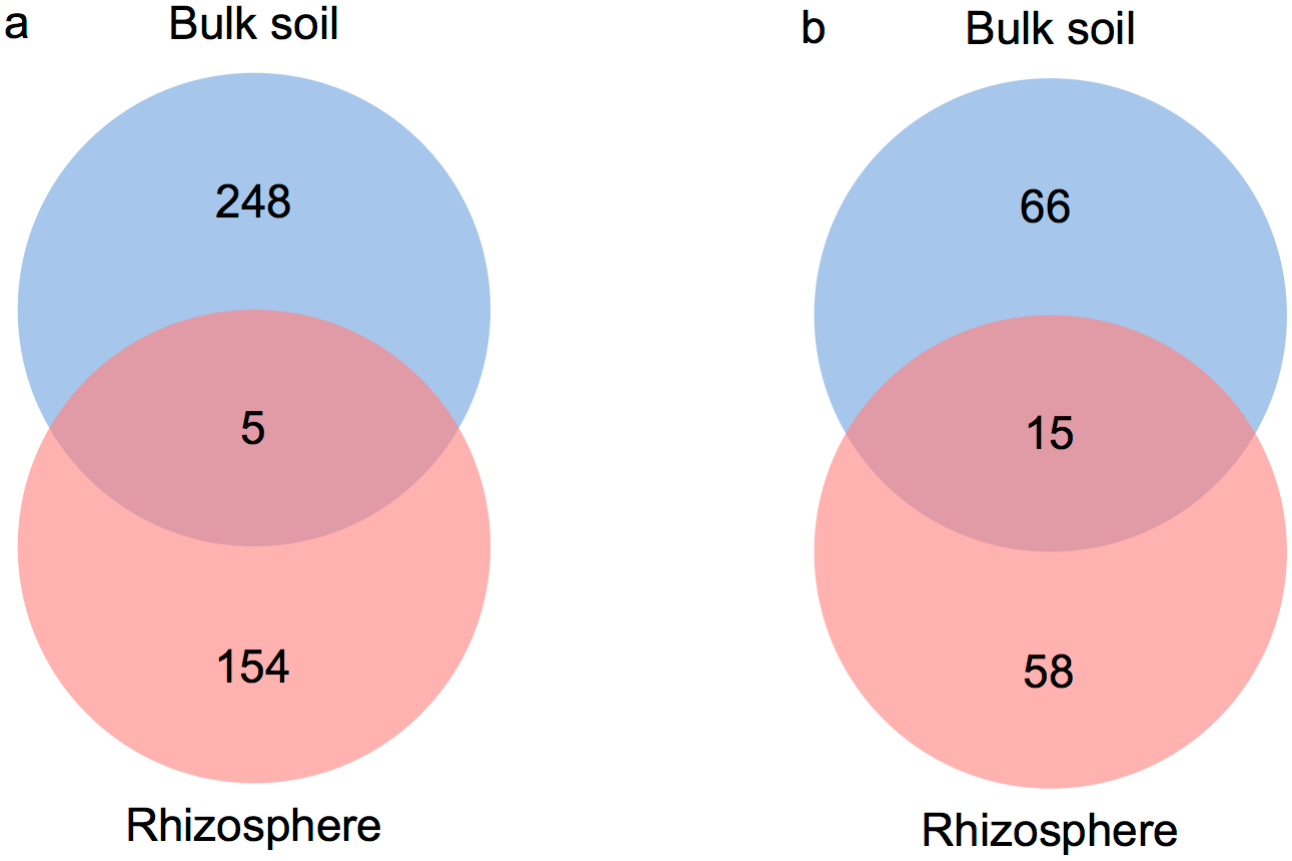
Venn diagram showing the number of shared phylotypes of (a) bacteria and (b) fungi between the rhizosphere and bulk soil communities.

An nMDS plot showed that bacterial and fungal communities were significantly different between bulk and rhizosphere soil samples (ANOSIM: R_bacteria_ = 0.8, R_fungi_ = 0.4; both P < 0.05), as measured by weighted UniFrac dissimilarities (Fig 3). This was supported using a Venn diagram (Fig 4a and 4b), as only 1% of the bacterial OTUs and 11% of the fungal OTUs, respectively, were shared between the bulk and the rhizosphere soil. On the basis of previous studies [7, 25–27] and the data presented above this is not an unexpected result. Differences in community composition are probably due to chemical differences between the two environments. Bulk soil has relatively low nutrient concentrations, whereas roots exude organic carbon and other nutrients [28]. This could explain, for example, why Protobacteria are more abundant in the rhizosphere, as many members of this phylum tend to dominate in environments where organic resources are more available [29]. Overall, these results suggest that *Welwitschia* strongly selects its rhizosphere microbiota.

Closer analysis of the rhizosphere samples showed that an average of 16.8% and 2.5% of the bacterial and fungal OTUs, respectively, were shared between any two of these communities. Furthermore, no fungal and only 6 bacterial OTUs (representing 38% of the reads) were consistently present across the samples (S4 Fig). These included *Nitratireductor*, *Steroidobacter*, *Pseudonocardia* and three *Phylobacteriaceae* OTUs. Overall, this indicates a larger degree of variability between the *Welwitschia* rhizosphere communities. This appears to be a common feature of host-associated microbial communities (see [30] and references therein) and appears to contradict the evidence for selectivity presented above. However, this apparent contradiction can be resolved assuming a competitive lottery model, applied to explain the variability of epiphytic bacterial community of the green alga *Ulva australis* [30, 31]. In this model microbial communities are hypothesized to occupy a certain niche (e.g. the rhizsosphere) based on the functions they perform; that is, microbial assemblages are based on functional guilds rather than species. Consequently, the set of species present in any rhizosphere community would be determined by which members of the guild, available in the surrounding soil, colonise the space available first. Future work with a larger number of samples, deeper sequencing and including functional attributes (genes) is needed to resolve whether microorganisms present in the *Welwitschia* root microbiome are recruited stochastically from the local soil community or actively based on the functions they perform.

It has been postulated that the ability of some desert plant species to survive under extreme conditions is linked to the fact that they associate with plant growth-promoting microbes [32, 33]. Several bacterial and fungal OTUs classified as belonging to plant-beneficial microbes were unique to or overrepresented in the rhizosphere of Welwitschia. These included, for instance, three different genera (i.e. *Bradyrhizobium*, *Ensifer* and *Mesorhizobium*) and three OTUs (family *Phylobacteriaceae*) of rhizobia, which have the ability to fix atmospheric nitrogen. *Acinetobacter* and *Sphingomonas*, known to solubilize soil-insoluble phosphate [34]. *Nitratireductor*, commonly reported as able to reduce nitrate to nitrite, that could be involved in nitrogen metabolism. *Steroidobacter*, recognised to produce brassinosteroids, which have been reported to control seed germination, stem and root elongation, vascular differentiation, leaf expansion and stress protection in plants [35]. *Pseudonocardia* as well as *Rhizophagus* were also found in the rhizosphere of *Welwitschia* plants. *Pseudonocardia* is well-known for producing antibiotic compounds [36], which can theoretically counteract some phytophatogenic microbes, whereas *Rhizophagus*, an arbuscular mycorrhizal fungus (AMF), could potentially supply phosphorus and other nutrients to *Welwitschia* in exchange for plant carbon [37]. It is noteworthy that *Nitratireductor*, *Steroidobacter*, *Pseudonocardia* and the three *Phylobacteriaceae* OTUs were core members of the rhizosphere community (see above). However, more research is needed to elucidate the role of the members of these microbial communities, as it is well known that plant growth promoting characteristics are strain dependent.

In conclusion, we have shown that the rhizosphere of *Welwitschia* harbours diverse and distinct bacterial and fungal communities compared to the bulk soil. Many of the genera consistently observed in the rhizosphere samples are known to contain strains with plant-growth promoting abilities. Further investigations using culture-based approaches will help in elucidating whether or notthese microbes interact synergistically to promote *Welwitschia* plant health and productivity.

## Supporting Information

S1 Fig *Welwitschia* plants dotted across an arid landscape (left). The exposed radial root system of a *Welwitschia* plant (right).
(TIF)

S2 Fig Rarefaction curves. a) bacteria, b) fungi. Sample nomenclature is as in S1 Table.
(PDF)

S3 Fig Bar graph showing the phylum-level distribution of (a) bacterial and (b) fungal OTUs (97% cutoff). The taxonomic affiliation was performed using the Ribosomal Database Project Classifier (bacteria) and the UNITE database (fungi).
(PDF)

S4 Fig Venn diagram showing the number of shared phylotypes observed between the rhizosphere samples. a) bacteria, b) fungi. Sample nomenclature is as in S1 Table.
(PDF)

S1 Table Bacterial diversity. Sample nomenclature indicates the sample type (S = bulk soil; R = rhizosphere), replicate (S = 1 to 5, R = 1 to 3) and pseudoreplicate (a, b).
(DOCX)

S2 Table Fungal diversity. Sample nomenclature is as in S1 Table.
(DOCX)
