# Supplementary figures and images for: Specific Microbial Communities Associate with the Rhizosphere of Welwitschia mirabilis, a Living Fossil
Source: PLoS One. 2016 Apr 11;11(4):e0153353. doi: 10.1371/journal.pone.0153353 (PMC4827806; doi:10.1371/journal.pone.0153353)

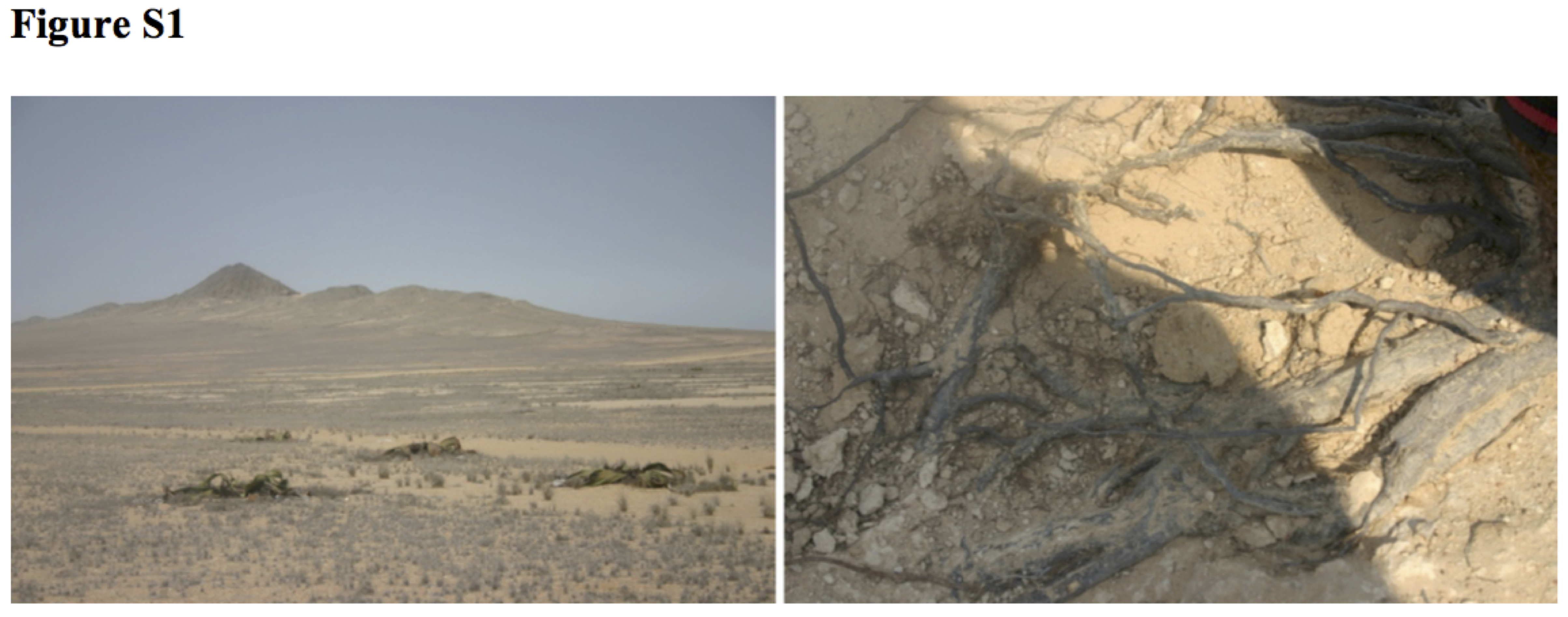

Supplement: S1 Fig — Welwitschia plants dotted across an arid landscape (left). The exposed radial root system of a Welwitschia plant (right). (TIF) [file pone.0153353.s001.tif]

S2 Figure

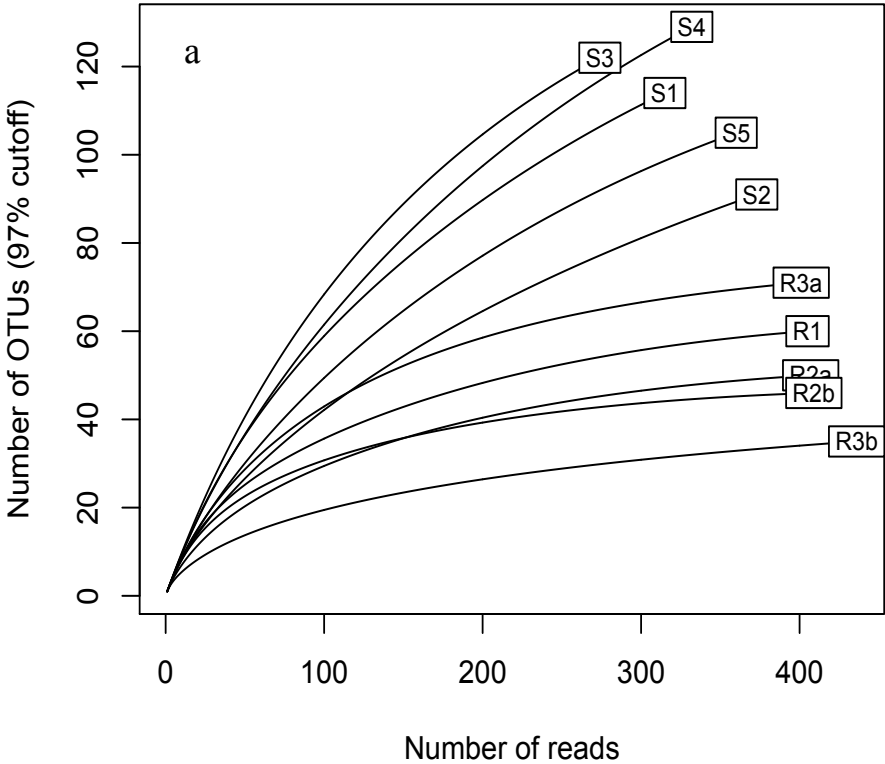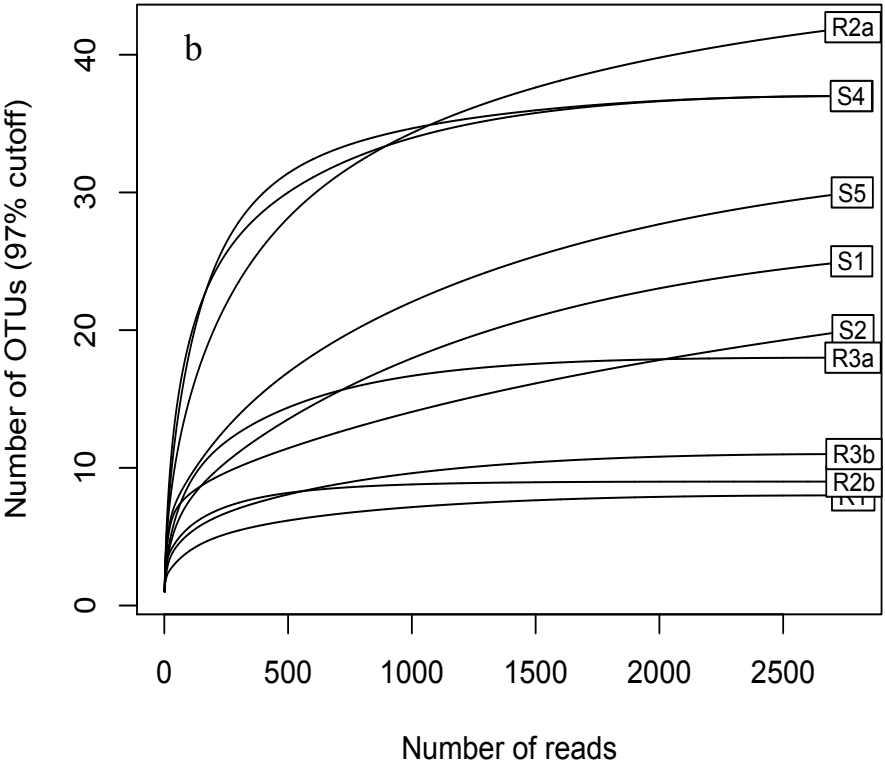

Supplement: S2 Fig — a) bacteria, b) fungi. Sample nomenclature is as in S1 Table. (PDF) [file pone.0153353.s002.pdf]

S3 Figure

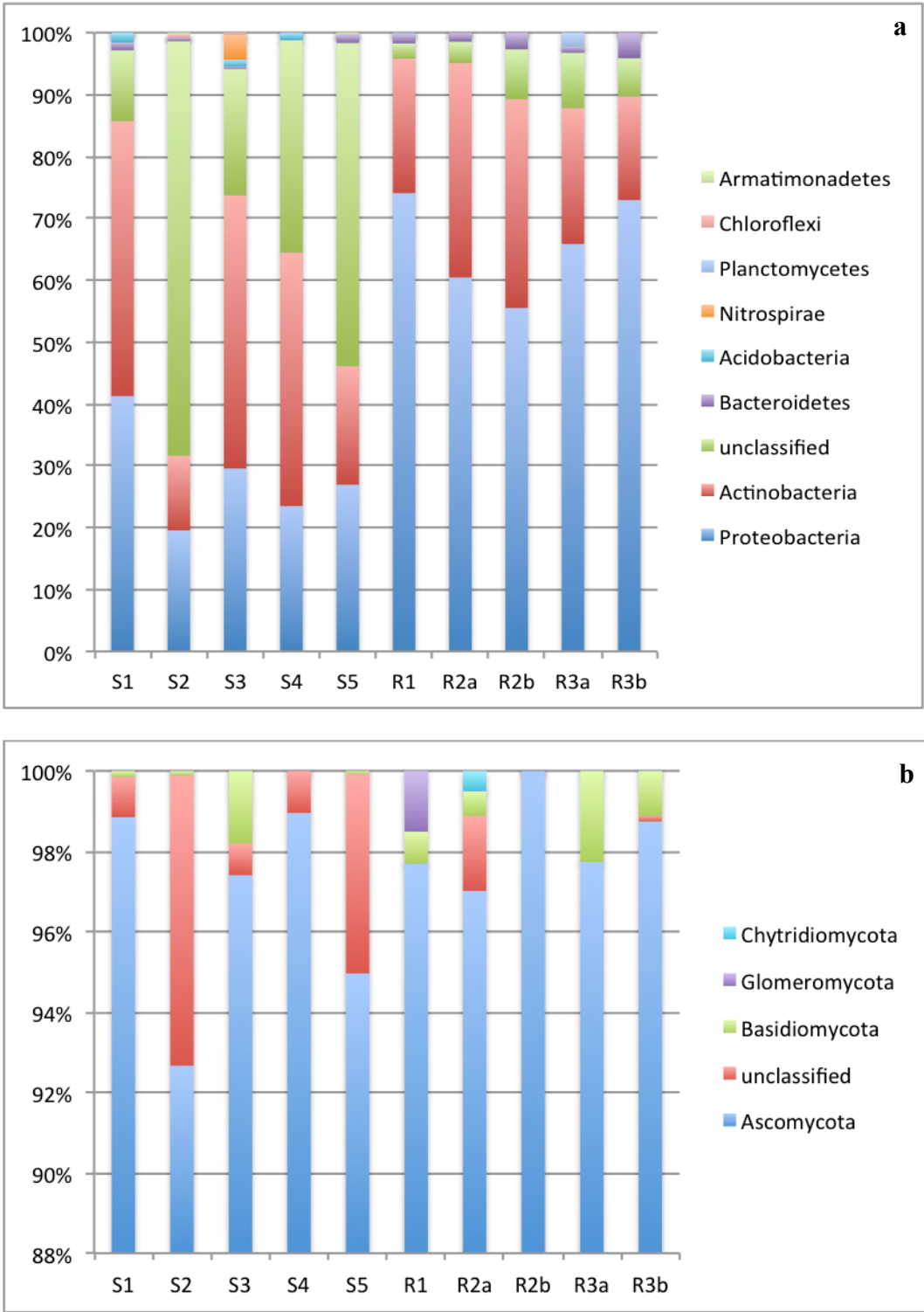

Supplement: S3 Fig — The taxonomic affiliation was performed using the Ribosomal Database Project Classifier (bacteria) and the UNITE database (fungi). (PDF) [file pone.0153353.s003.pdf]

S4 Figure

a

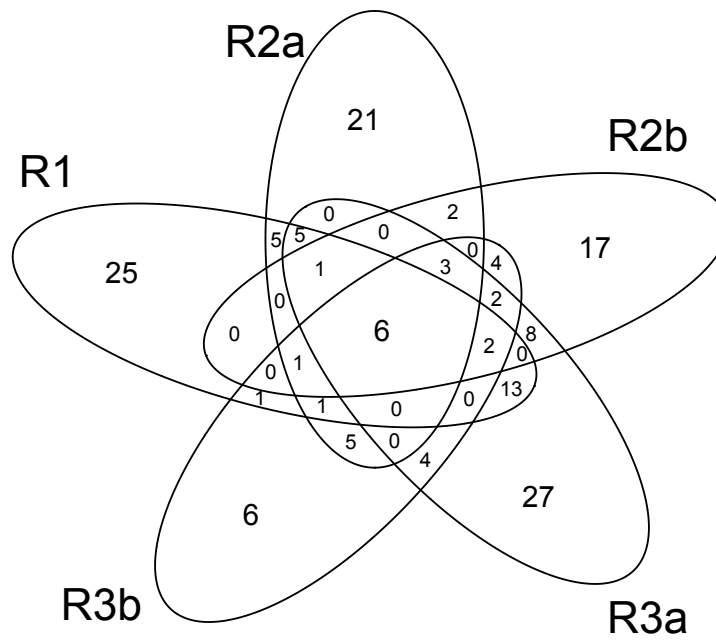

b

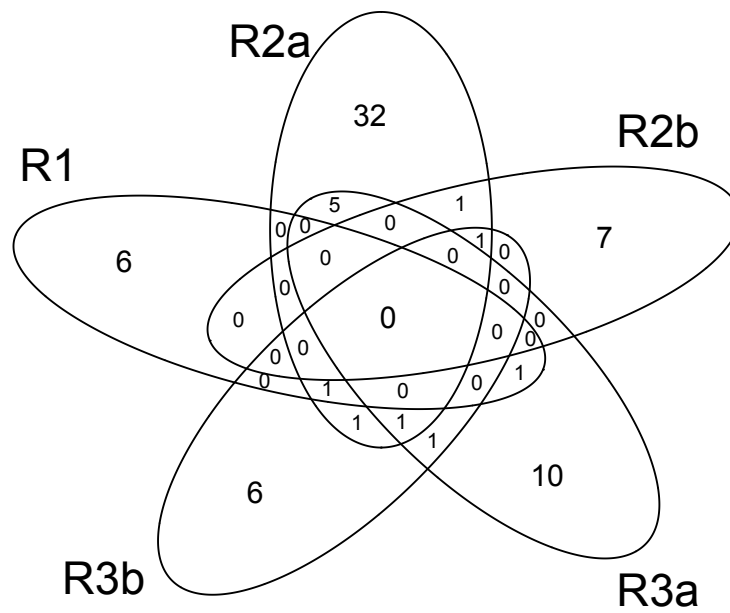

Supplement: S4 Fig — a) bacteria, b) fungi. Sample nomenclature is as in S1 Table. (PDF) [file pone.0153353.s004.pdf]
