# Supplementary material for: Specific Microbial Communities Associate with the Rhizosphere of Welwitschia mirabilis, a Living Fossil
Source: PLoS One. 2016 Apr 11;11(4):e0153353. doi: 10.1371/journal.pone.0153353 (PMC4827806; doi:10.1371/journal.pone.0153353)
Supplement: S1 Table — Sample nomenclature indicates the sample type (S = bulk soil; R = rhizosphere), replicate (S = 1 to 5, R = 1 to 3) and pseudoreplicate (a, b). (DOCX) [file pone.0153353.s005.docx]

**S1 Table. Bacterial diversity**

| **Sample** | **Richness** | **Shannon** | **Simpson** | **Inverse**  **Simpson** | **Pielou's**  **evenness** | **Good’s**  **coverage** |
| --- | --- | --- | --- | --- | --- | --- |
| S1 | 114 | 4.32 | 0.98 | 52.09 | 77.90 | 82 |
| S2 | 91 | 3.59 | 0.94 | 17.44 | 71.13 | 87 |
| S3 | 122 | 4.57 | 0.99 | 75.99 | 80.32 | 80 |
| S4 | 129 | 4.38 | 0.98 | 44.99 | 87.34 | 81 |
| S5 | 105 | 3.86 | 0.95 | 20.40 | 77.76 | 87 |
| *Mean ± SE* | *112.2±6.6^a^* | *4.1±0.2^a^* | *1.0±0.01^a^* | *42.2±11^a^* | *78.9±2.6^a^* |  |
|  |  |  |  |  |  |  |
| R1 | 60 | 3.49 | 0.96 | 22.88 | 48.02 | 87 |
| R2a | 50 | 2.91 | 0.88 | 8.11 | 46.86 | 97 |
| R2b | 46 | 3.25 | 0.94 | 17.85 | 39.01 | 98 |
| R3a | 71 | 3.71 | 0.96 | 24.58 | 54.16 | 97 |
| R3b | 35 | 2.19 | 0.77 | 4.43 | 44.62 | 97 |
| *Mean ± SE* | *52.4±6.1^b^* | *3.1±0.3^b^* | *0.9±0.03^a^* | *15.6±4^b^* | *46±2.4^b^* |  |

Different letters indicate that the differences in mean between rhizosphere (R) and bulk soil (S) samples were significant (Paired t-test, P<0.05).
