# Supplementary material for: Specific Microbial Communities Associate with the Rhizosphere of Welwitschia mirabilis, a Living Fossil
Source: PLoS One. 2016 Apr 11;11(4):e0153353. doi: 10.1371/journal.pone.0153353 (PMC4827806; doi:10.1371/journal.pone.0153353)
Supplement: S2 Table — Sample nomenclature is as in S1 Table. (DOCX) [file pone.0153353.s006.docx]

**S2Table. Fungal diversity**

| **Sample** | **Richness** | **Shannon** | **Simpson** | **Inverse**  **Simpson** | **Pielou's**  **evenness** | **Good’s**  **coverage** |
| --- | --- | --- | --- | --- | --- | --- |
| S1 | 25 | 1.05 | 0.46 | 1.86 | 46.85 | 99 |
| S2 | 20 | 1.48 | 0.65 | 2.84 | 51.42 | 99 |
| S3 | 37 | 2.41 | 0.83 | 5.94 | 42.15 | 100 |
| S4 | 37 | 2.16 | 0.76 | 4.18 | 48.14 | 100 |
| S5 | 30 | 1.68 | 0.73 | 3.66 | 58.14 | 100 |
| *Mean ± SE* | *29.8±3.3* | *1.7±0.2* | *0.7±0.1* | *3.7±0.7* | *49.3±2.6* |  |
|  |  |  |  |  |  |  |
| R1 | 8 | 0.63 | 0.34 | 1.51 | 17.40 | 100 |
| R2a | 42 | 2.08 | 0.81 | 5.14 | 57.36 | 99 |
| R2b | 9 | 1.16 | 0.60 | 2.53 | 59.35 | 100 |
| R3a | 18 | 1.29 | 0.59 | 2.43 | 71.47 | 100 |
| R3b | 11 | 0.79 | 0.37 | 1.60 | 47.64 | 100 |
| *Mean ± SE* | *17.6±6.3* | *1.2±0.2* | *0.5±0.1* | *2.6±0.6* | *50.6±9.1* |  |

Sample nomenclature indicates the sample type (S = bulk soil; R = rhizosphere), replicate (S = 1 to 5, R = 1 to 3) and pseudoreplicate (a, b). No differences in means were detected between R and S samples.
